# The One Who Went Free

**Published:** 1914-01

**Authors:** Gertrude Seymour


### The One Who Went Free.

BY GERTRUDE SEYMOUR.

Alone in the Union Station, Chicago, stood a woman. Her dress of heavy mourning emphasized the drawn pallor of her face and the frightened expression of her wide eyes. Other passengers who had come in on that morning train had taken their way from the station; but she stood there in the waiting room, hesitating. She seemed to be waiting not so much for some one to meet her, but she was uncertain as to which way to go.

"Can I help you?" I said as I approached her. "You seem to be alone." How she started! But presently the terror died down in her eyes, though she made no reply. "I am here to help travelers," I continued. "Where do you want to go?"

From a dingy bag she drew out a telegram: "Come at once. Marguerite ill." The address given was of a street miles away on the South Side.

"Will he let me have her, do you think?" she questioned incoherently. What could that mean? The woman's face was quivering; her self-control was giving away.

"We shall go and see her," I answered. "But first you must have some coffee, for we have a long distance to go."

Nimbly she followed me into the dining room; and as we had our coffee in a quiet corner she gasped out her story, searching my face from time to time to make sure that she was safe in trusting me.

She had married a man sixteen years ago, trusting him utterly, happy in the prospect of her own home and the hoped for children. A year later Marguerite came, and in the remembrance of that joy the lines in her face softened for a moment, but only for a moment; the sorrow returned. The second year her husband grew busier, was later at the office, and often the firm sent him out of town for several days. She was lonely those days while another baby was coming. Her husband was away a great deal. "And I said nothing, for I wanted him to feel free to come and go," she added with a gesture of pride.

The second child was restless and fretful, not sunny-tempered like Marguerite. It was often dull, and seemed not to see well. Another year, and then suddenly all the romance in her life was shattered. When she told her husband of the promise of a third child, he threw reserve to the winds. He told her brutally that he could afford no more children, and that this one must be prevented. She scarcely survived the criminal operation; but as soon as she was strong enough to walk she took the year-old boy to a good doctor.

"I'd hid my fears in my heart about Tom," she sobbed, "though I couldn't help being suspicious sometimes when he went away so much. Then I'd hate myself for thinking such things. But now the doctor told me. He saw that I was after the truth, and he told me that my boy would be blind because his father was a bad man." She covered her face for a moment, then she went on rapidly. "Tom acted something awful," she gasped. "Then he took Marguerite and came to Chicago, and wouldn't let me see her or hear from her any more. O, why do you think he wants me now? Do you suppose that she is dead?" And in a spasm of emotion she half rose from the table. I rose too, and we went to our car silently. Seated there, she gazed stupidly at the changing scenes, dulled by the noise of traffic. I roused her by asking: "Why did he take Marguerite from you?" "He said I wasn't fit to have her," she replied dully. "I reckon I wasn't. When I knew what the matter was with baby, and thought that later it might come to Marguerite, I thought we'd better all just die. So I gave the children some poison and took a lot myself. Tom was away."

When I could speak I said: "And they could save only you and the little girl?"

"Yes'm. The boy died. They put me in an asylum for a year, for I went out of my head. But when I got well they turned me out again."

We were still for a long time. Just before we reached our street I gave her a card with my address, that she might know how to find me if she wanted me again. It was well that I did so, for at the door of the flat we were parted. In answer to our ring, a tall man, handsome according to the cheap type, came to the door. Her cry, "O Tom!" was hardly more than a sob. A harsh charge not to be a fool, a refusal with scant courtesy to amit me, a "meddling missionary woman," and the door was locked in my face.

Only later, when the mother, penniless and with her fifteen-year-old daughter to care for, came to us for further guidance did I learn the rest of that story. Left to her own devices, practically alone in Chicago, little Marguerite had been won by promises of pretty things to her own undoing. Her father knew of but one remedy for such a condition. He paid a "doctor" to rent the flat to which we had gone and to perform there in due time the unspeakable operation upon the helpless girl.

There was a law for those who rented the flat. There was a law for the "doctor" who did the deed. But the man—the father to whose account were charged two abortions, three poisonings, a blind baby, a case of insanity, and a girl robbed of her girlhood—for him there was no law; he was the one who went free.—*Exchange.*

---

X-ray plates, properly interpreted, are of great service in the diagnosis of mastoiditis, acute and chronic. Stereoscopy and comparison of the pictures of the two sides enhance the value of the radiographic examination.—*American Journal of Surgery.*

---

Mrs. Malaprop—Young Shap will have to apologize before I speak to him again.

Miss Interest—Did he insult you?

Mrs. Malaprop—Did he? The last time I met him I told him that my uncle had locomotive ataxia, and he asked me if he "whisteled at crossings."

---

A Fable Love Story: At the approach of Valentine Day last year I offered a prize of $5.00 to the little boys of my Sunday school class for the best short love story. I have one of the stories here, and I am going to read it to you:

Mr. McWade then read:

"A poor man fell in love with a lady whose mother was a rich toy dealer. The poor man could not marry the rich lady because he had no money. A villain then offered him $50.00 if he would become a drunkard. The poor man wanted the money to get married with, so he agreed; but when he got to the beer saloon, he said: 'No, I will not become a drunkard, even for great riches.' On the way home, he found a bag of gold. So the young lady married him. It was a splendid wedding and the next day they had twins."

"Moral: Virtue is its own reward."—*Exchange.*

